# Coexistence of Acute Appendicitis and Mesenteric Cystic Lymphatic Malformation in an Adult: A Case Report and Narrative Review of Intraoperative Management Strategies

**DOI:** 10.3390/life15091390

**Published:** 2025-09-01

**Authors:** Laurențiu Augustus Barbu, Liliana Cercelaru, Ionică-Daniel Vîlcea, Valeriu Șurlin, Stelian-Stefaniță Mogoantă, Tiberiu Stefăniță Țenea Cojan, Nicolae-Dragoș Mărgăritescu, Mihai Popescu, Gabriel Florin Răzvan Mogoș, Liviu Vasile

**Affiliations:** 1 Department of Surgery, Railway Clinical Hospital Craiova, University of Medicine and Pharmacy of Craiova, 2 Petru Rares Street, 200349 Craiova, Romania; laurentiu.barbu@umfcv.ro (L.A.B.); tiberiu.tenea@umfcv.ro (T.S.Ț.C.); 2 Department of Embryology and Anatomy, University of Medicine and Pharmacy of Craiova, 200349 Craiova, Romania; liliana.cercelaru@umfcv.ro; 3 Department of Surgery, Emergency County Hospital, University of Medicine and Pharmacy of Craiova, 2 Petru Rares Street, 200349 Craiova, Romania; ionica.vilcea@umfcv.ro (I.-D.V.); vsurlin@gmail.com (V.Ș.); ssmogo@yahoo.com (S.-S.M.); dmargaritescu@yahoo.com (N.-D.M.); vliviu777@yahoo.com (L.V.); 4 Imaging Department, University of Medicine and Pharmacy of Craiova, 200349 Craiova, Romania; mihai_popescu_rad@yahoo.com

**Keywords:** mesenteric cystic lymphatic malformations, acute appendicitis, intraoperative management, segmental enterectomy, surgical algorithm, lymphatic malformations

## Abstract

**Background**: Mesenteric cystic lymphatic malformations (MCLMs) are rare benign lymphatic malformations predominantly diagnosed in children. Adult cases are exceptional and typically discovered incidentally during imaging or surgery for unrelated conditions. Their intraoperative identification, particularly in emergency settings, poses diagnostic and surgical challenges due to anatomical complexity and potential vascular involvement. **Methods**: A literature review was performed in PubMed and Scopus to contextualize this case, focusing on intraoperative management strategies, recurrence risk, and surgical decision-making in mesenteric lymphatic malformations. Case reports, case series, and reviews in English with relevant clinical and surgical data were included, while duplicates, non-English publications, abstracts without full text, and studies lacking essential information were excluded. **Case Presentation**: We report a 45-year-old male who presented with acute right lower quadrant pain, clinically and radiologically consistent with acute appendicitis. Contrast-enhanced CT incidentally identified a mesenteric cystic lesion near the terminal ileum. Intraoperative findings confirmed phlegmonous appendicitis coexisting with a large MCLM, requiring segmental enterectomy and appendectomy. Histopathology confirmed the diagnosis of MCLMs. **Conclusions**: This case highlights the rare coexistence of acute appendicitis and mesenteric lymphatic malformations in an adult, illustrating the surgical challenges of unexpected lymphatic lesions in emergency settings. Emphasizing real-time intraoperative decision-making, we propose an anatomy-driven algorithm that balances complete excision with safer, conservative options based on lesion features, surgical risk, and multidisciplinary input.

## 1. Introduction

Mesenteric cystic lymphatic malformations are exceedingly rare benign malformations of lymphatic origin, with an estimated incidence of less than 1 per 100,000 hospital admissions and under 200 cases reported globally [1]. These lesions are more commonly diagnosed in pediatric populations, reflecting their congenital nature; however, adult presentations, though exceptional, have been documented and typically occur as incidental findings during imaging or intraoperative exploration for unrelated abdominal pathologies [2,3].

In adults, MCLMs are often asymptomatic and do not typically present a diagnostic dilemma preoperatively. Instead, their identification poses an intraoperative management challenge, particularly when discovered during emergency procedures such as appendectomies. Decision-making in such scenarios must be individualized, taking into account lesion size, anatomical location, patient stability, and the potential risk of future complications [4,5].

Although preoperative imaging with ultrasound, contrast-enhanced CT, or MRI can aid in detection, these modalities often lack specificity for rare lymphatic lesions, leading to delayed or intraoperative diagnosis [1,4]. Histopathologic confirmation remains the gold standard, relying on lymphatic markers such as Prox1, CD31, and D2-40; however, DNA-mutation analysis can further support the diagnosis, as somatic activating mutations in the *PIK3CA* gene—and less frequently in *AKT1* or *NRAS*—have been reported in lymphatic malformations, highlighting that histology alone is not always sufficient [2,6,7].

Clinically, mesenteric cystic lymphatic malformations can present with a broad spectrum of manifestations. Many patients remain asymptomatic, with lesions discovered incidentally during imaging or surgery for unrelated conditions. Symptomatic cases may develop nonspecific abdominal pain, distension, nausea, vomiting, or a palpable abdominal mass. Larger lesions can exert a mass effect on adjacent structures, occasionally leading to bowel obstruction, volvulus, intracystic hemorrhage, or secondary infection. Rarely, acute abdominal presentations have been reported, emphasizing the variable and sometimes deceptive clinical profile of these malformations [1].

This case offers a unique opportunity to address the gap between preoperative diagnostic limitations and real-time surgical decision-making in rare lymphatic lesions.

Therefore, we present a rare case of an adult patient undergoing emergency appendectomy in whom a mesenteric cystic lymphatic malformation was incidentally discovered. In contrast to prior reports that focus primarily on diagnosis, this article emphasizes intraoperative management. By combining a case report with a narrative literature review, we propose a structured surgical algorithm for managing such findings when encountered unexpectedly, underscoring the importance of anatomical assessment and surgical adaptability in emergent settings.

## 2. Case Presentation

### 2.1. Patient Information

A 45-year-old male with no significant past medical history and no prior abdominal surgeries presented to the Emergency Department with acute onset right lower quadrant pain. The pain had lasted less than 48 h and was accompanied by nausea and multiple episodes of non-bilious vomiting. He reported no history of previous similar episodes, no weight loss, and no bowel or urinary complaints.

### 2.2. Clinical Findings

On physical examination, the patient was afebrile but appeared mildly dehydrated. Abdominal examination revealed tenderness and guarding in the right lower quadrant, with Rovsing’s sign (contra-lateral rebound tenderness) and Blumberg’s sign (rebound tenderness). No palpable masses were noted. Digital rectal examination was normal.

### 2.3. Timeline

A structured timeline of the patient’s presentation, diagnostic workup, surgical intervention, and recovery is provided to illustrate the progression and management of the case (Table 1).

### 2.4. Diagnostic Assessment

Laboratory investigations revealed leukocytosis with marked neutrophilia, elevated urea, C-reactive protein, and fibrinogen levels—findings suggestive of an acute inflammatory or infectious process with possible prerenal azotemia (Table 2).

**Figure 1:**
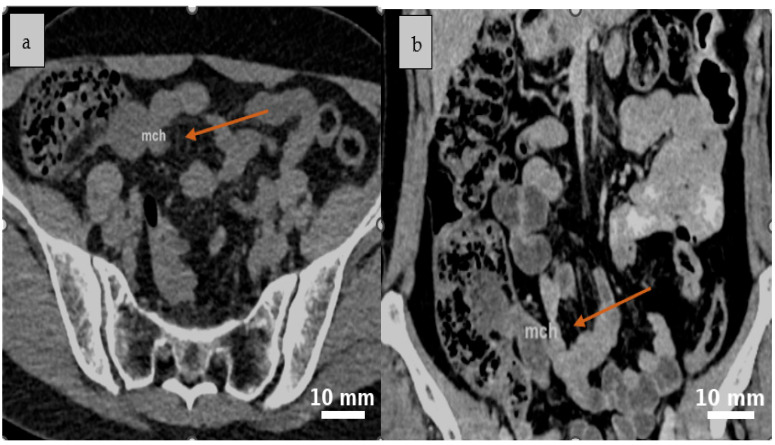
A well-defined, round to oval cystic lesion with fluid attenuation is identified in the ileocecal region on abdominal CT scan native (**a**) and confirmed on post-contrast coronal reconstruction, consistent with a mesenteric cyst (**b**). Yellow arrows indicate the cystic lesion (*mch = mesenteric cystic hygroma—lymphatic malformation*).

**Figure 2:**
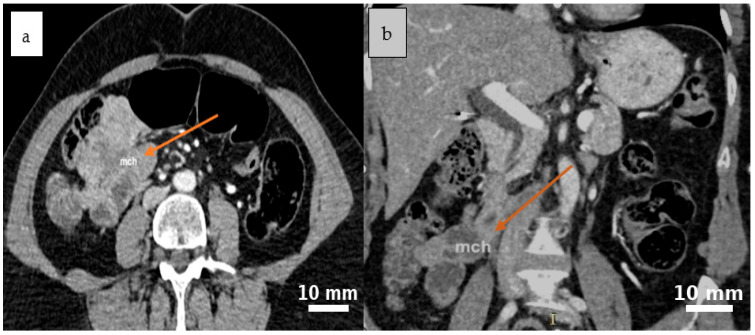
Post-contrast CT scan reveals a cystic lesion situated between the intestinal loops in the ileocecal region, characterized by mild peripheral attachment and slightly heterogeneous internal content, as seen in the axial (**a**) and coronal (**b**) planes. Yellow arrows indicate the cystic lesion (*mch = mesenteric cystic hygroma—lymphatic malformation*).

Abdominal contrast-enhanced CT (native and post-contrast axial and coronal reconstructions) demonstrated a well-defined, round-to-oval cystic lesion in the ileocecal region with fluid attenuation, subtle peripheral attachment, and mildly heterogeneous content, suggestive of a mesenteric cyst (Figure 1 and Figure 2).

### 2.5. Therapeutic Intervention

The patient underwent a midline laparotomy under general anesthesia. Intraoperative exploration confirmed a descending mediocecal appendix approximately 3.5 cm × 1.5 cm × 1.3 cm in size, covered partially by membranous exudate and surrounded by serous fluid—consistent with acute phlegmonous appendicitis.

Further mobilization of the distal 10–15 cm of the terminal ileum revealed multiple mesenteric cystic lesions, the largest measuring approximately 8 cm, incompletely septated and closely adherent to the adjacent bowel. On dissection, the cyst demonstrated a smooth internal surface with delicate digitiform projections into the surrounding adipose tissue, which showed focal yellowish punctate areas.

**Figure 3:**
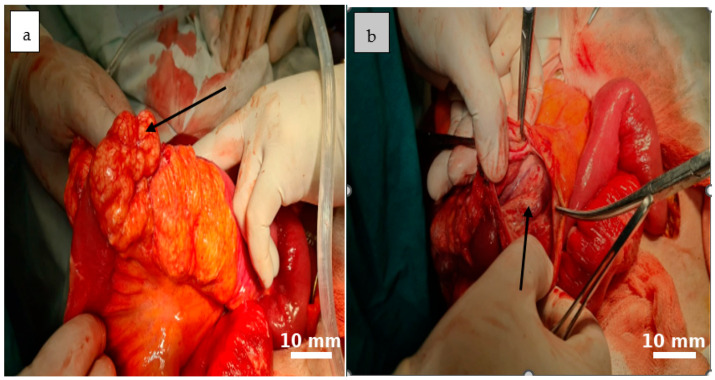
An irregularly lobulated cystic lesion is observed within the mesenteric tissue ((**a**), black arrow). The cyst cavity appears tense and well-vascularized, with a reddish-orange hue that contrasts distinctly with the adjacent intestinal structures ((**b**), black arrow).

**Figure 4:**
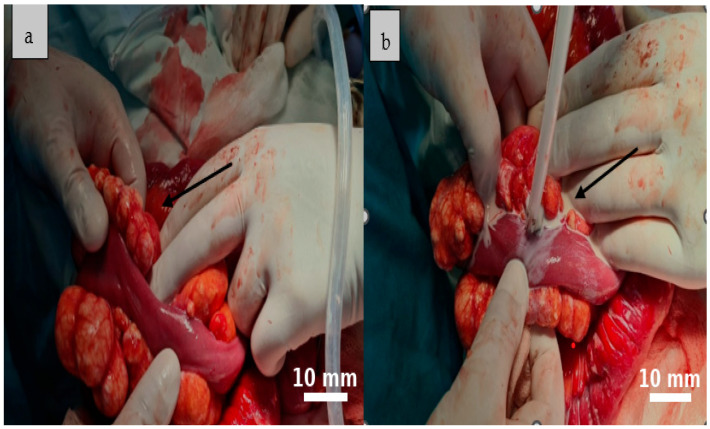
The cystic formations identified during surgery are consistent with multiple mesenteric lymphatic cysts. Their multilobulated appearance, tense walls, and prominent vascularization suggest a benign origin, though with potential for considerable mass effect ((**a**), black arrow). The cysts contained a characteristic milky fluid, typical of lymphatic content ((**b**), black arrow).

Gentle compression of the cyst released milky, chylous fluid (Figure 3 and Figure 4).

**Figure 5:**
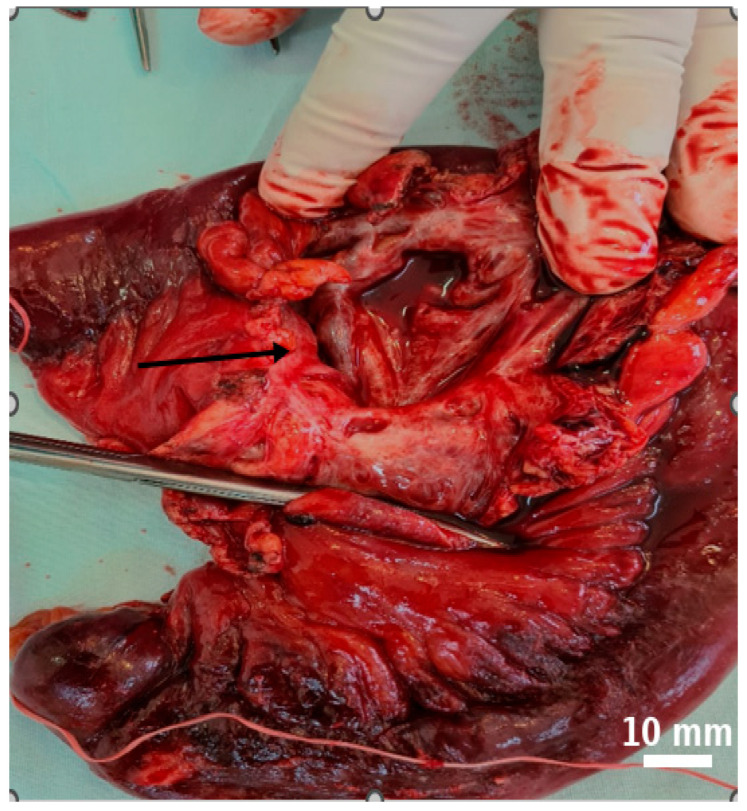
The segmental enterectomy specimen shows multiple cystic areas in the mesentery, which did not affect the vascularization. Black arrow indicates the cystic lesions within the resected specimen.

A total excision of the largest mesenteric cyst was performed via segmental enterectomy, followed by a side-to-side enteroenteric anastomosis (Figure 5). The inflamed appendix was removed simultaneously.

Due to dense adhesions and involvement of the terminal ileum, open segmental enterectomy was considered the safest option in this emergency setting, allowing for complete excision of the lesion with histologically clear margins.

### 2.6. Follow-Up and Outcomes

The patient’s postoperative course was uneventful. He tolerated oral intake by postoperative day 2, completed intravenous antibiotics, and was discharged in stable condition on postoperative day 5.

### 2.7. Histopathological Findings

**Figure 6:**
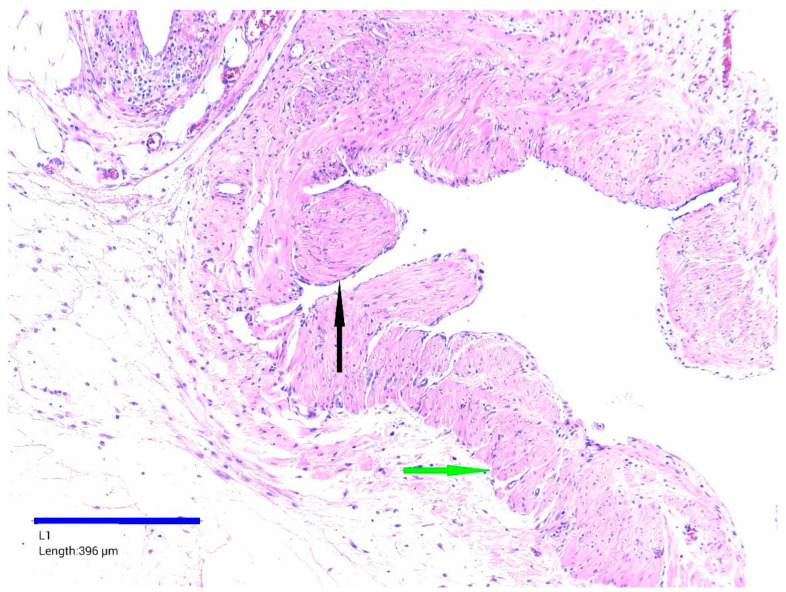
Mesenteric cyst wall, HE staining, ×100. Flattened epithelium lining—black arrow, bundles of smooth muscle—green arrow.

Microscopic examination of the resected small intestine revealed multiple cystic structures of various sizes and shapes within the mesenteric tissue. The cysts had variably thickened fibrous walls, focal chronic inflammatory cell infiltrate, dilated and hyperemic blood vessels, and small foci of hemorrhage. In thicker-walled cysts, well-defined bundles of smooth muscle fibers were also identified (Figure 6).

Most cysts showed a flattened or denuded epithelial lining. Focal areas of epithelial proliferation were observed forming delicate intraluminal pseudopapillary structures, without cellular atypia. The lumens were largely empty, though some contained amorphous eosinophilic material or scattered erythrocytes. Final histopathological diagnosis confirmed a mesenteric cystic lymphatic malformation.

## 3. Discussion

### 3.1. Epidemiology and Etiopathogenesis of Mesenteric Cystic Lymphatic Malformations

#### 3.1.1. Rarity and Clinical Significance

Mesenteric cystic lymphatic malformations are rare benign malformations of the lymphatic system, with approximately 200 cases reported in the literature [8,9]. They may develop in various intra-abdominal locations, including the small bowel mesentery, retroperitoneum, gastrointestinal tract, and visceral organs [10]. Although histologically benign, their size and proximity to vital structures can complicate surgical management, particularly when discovered incidentally during emergency interventions—as in our case.

#### 3.1.2. Pediatric and Adult Presentations

MCLMs are most commonly diagnosed in children, reflecting their congenital origin as developmental lymphatic anomalies. In large pediatric series, abdominal lymphatic malformations account for roughly 9% of cases [9,11]. While often asymptomatic, larger lesions may exert mass effect, potentially causing bowel obstruction, volvulus, or intracystic hemorrhage [10,12].

In adults, MCLMs may arise secondary to lymphatic obstruction from prior abdominal surgery, trauma, chronic inflammation, or radiation [12]. Therefore, both congenital and acquired forms should be considered during surgical planning and risk assessment. In our patient, the presence of acute appendicitis may have acted as an acute inflammatory trigger, possibly contributing to the lesion’s size.

#### 3.1.3. Pathogenesis and Recurrence Risk

Although the precise pathophysiology of lymphatic malformations is not yet fully elucidated, emerging evidence indicates that somatic DNA mutations leading to mosaicism play a significant role in their development [13]. This genetic basis helps explain the tendency for recurrence even after technically complete excision and supports the need for a multidisciplinary treatment approach, potentially involving surgeons, interventional radiologists, and medical specialists, with both surgical and non-surgical modalities—including sclerotherapy or targeted medical therapies—as part of long-term patient care.

#### 3.1.4. Intraoperative Considerations

Lesions located within the jejunal mesentery appear more likely to require enterectomy, a finding plausibly attributable to the region’s intricate vascular anatomy and the limited availability of surgical margins [3]. This observation underscores the clinical heterogeneity of mesenteric lymphatic malformations (MLMs) and emphasizes the importance of tailoring surgical management to intraoperative findings rather than relying solely on preoperative imaging. Our case exemplifies this principle within the context of emergency surgery, where the operative plan was individualized based on intraoperative assessment.

#### 3.1.5. Possible Interaction with Acute Inflammation

In the present case, it is plausible that the size of the MCLMs was influenced by the concurrent acute appendicitis. Given that lymphatic malformations consist of lymphatic tissue, it is conceivable that the inflammatory response triggered by infectious agents could have led to local swelling or increased cyst volume—a phenomenon rarely described in the literature but pathophysiologically plausible [9].

### 3.2. Clinical and Imaging Features

#### 3.2.1. Incidental Discovery in Emergency Settings

Although uncommon, intra-abdominal mesenteric lesions—both benign and malignant—are sometimes discovered incidentally during imaging or emergency surgery for unrelated conditions such as acute appendicitis. In our case, the mesenteric cystic lymphatic malformation was identified unexpectedly during emergency surgery performed for acute appendicitis. This scenario highlights a key challenge: while MCLMs are frequently asymptomatic, they may remain undetected until they cause a mass effect or are encountered intraoperatively. Prompt and individualized surgical decision-making is therefore essential, guided by the lesion’s morphology, anatomical relationships, and the patient’s overall clinical status.

#### 3.2.2. Differential Diagnosis

Benign mesenteric tumors such as desmoid fibromatoses, lipomas, and gastrointestinal stromal tumors (GISTs) can present with nonspecific abdominal symptoms caused by compression of adjacent structures [14,15,16]. By contrast, MCLMs—congenital lymphatic malformations—can reach considerable size while remaining clinically silent. They are most often detected incidentally, either during cross-sectional imaging or at the time of surgery, rather than presenting with overt clinical signs [17].

#### 3.2.3. Role and Limitations of Imaging

Modern imaging modalities—including ultrasound, computed tomography (CT), and magnetic resonance imaging (MRI)—have greatly enhanced the evaluation of patients presenting with right iliac fossa (RIF) pain. In acute settings, CT is the preferred modality due to its rapid availability and high anatomical precision, enabling accurate diagnosis of common conditions such as appendicitis and occasionally revealing incidental findings that may influence surgical strategy [18,19]. However, its specificity for rare cystic lesions such as MCLMs remains limited. Ultrasound, while widely accessible and non-invasive, is highly operator-dependent. MRI offers superior soft tissue contrast but is seldom used in emergency contexts because of time constraints and limited availability [19].

In our patient, a CT performed for suspected acute appendicitis did not clearly identify the MCLMs preoperatively, illustrating the limitations of imaging in such rare lesions and reinforcing the importance of intraoperative assessment.

### 3.3. Incidental Detection and Diagnostic Pitfalls

#### 3.3.1. Incidental Discovery of Mesenteric Lesions

Although rare, mesenteric lesions—both benign and malignant—are frequently discovered incidentally during imaging or intraoperative evaluation for unrelated abdominal conditions. Benign tumors such as desmoid fibromatoses, mesenteric lipomas, and gastrointestinal stromal tumors (GISTs) may cause vague or nonspecific symptoms due to mass effect or compression of adjacent structures [14,15,16,19,20].

#### 3.3.2. Mesenteric Cystic Lymphatic Malformations in Context

Among these lesions, mesenteric cystic lymphatic malformations—congenital malformations of the lymphatic system—are exceptionally uncommon and typically asymptomatic. Despite their potential to reach considerable size, they are most often detected incidentally during cross-sectional imaging or surgical exploration rather than presenting with acute symptoms [16,17]. In our patient, the lesion was not suspected preoperatively and was identified unexpectedly during emergency surgery for acute appendicitis.

#### 3.3.3. Implications for Emergency Surgery

In acute scenarios such as appendectomy, the incidental discovery of a mesenteric cyst necessitates a tailored intraoperative approach that accounts for lesion morphology, anatomical involvement, and patient stability. Our case illustrates the importance of prompt, situation-specific surgical decision-making to minimize complications and achieve optimal outcomes.

### 3.4. Surgical Management and Operative Strategies

#### 3.4.1. Surgical Classification of Intra-Abdominal Lymphatic Malformations

Intra-abdominal lymphatic malformations can be classified surgically according to their anatomical location and degree of structural involvement, a categorization that guides operative planning [7,18,19,20,21,22,23]. Four main types have been described: (1) pedunculated, mobile lesions amenable to simple excision; (2) sessile lesions involving mesenteric vasculature, which may require segmental bowel resection—as in the present case; (3) retroperitoneal forms potentially involving critical vascular or pancreatic structures; and (4) extensive infiltrative variants spanning multiple compartments, which present considerable technical challenges.

#### 3.4.2. Role of Surgical Exploration in Emergency Settings

When preoperative imaging is inconclusive, timely surgical exploration can help avoid delays in diagnosis and reduce morbidity [24,25,26,27]. Intraoperative decision-making should integrate clinical findings, anatomical relationships, and procedural feasibility. In emergency settings, the priority remains the safe and effective management of any incidental lesion, with flexibility to adapt the surgical plan to real-time findings [28,29].

#### 3.4.3. Extent of Resection and Patient-Specific Considerations

Complete surgical excision is generally recommended as the definitive treatment, especially due to the high recurrence rates associated with partial resection, marsupialization, or aspiration [1,7,30]. However, when lesions are firmly adherent to adjacent bowel or mesenteric vessels, segmental bowel resection may be required to ensure safe and complete removal. Although complete surgical excision is associated with low recurrence rates and favorable long-term outcomes in many series [30], this approach should be considered within a patient-specific risk–benefit framework. In cases where dense adhesions to mesenteric vessels or bowel are present, segmental resection with primary anastomosis may be appropriate, but only if it can be performed safely while preserving bowel function [20].

#### 3.4.4. Surgical Indications and Non-Surgical Alternatives

Large mesenteric lymphatic malformations can cause mass effect on adjacent bowel or vessels, increasing the risk of obstruction or ischemia. In such circumstances, surgery is often indicated. However, non-surgical approaches—including image-guided aspiration, sclerotherapy, or careful surveillance—may be appropriate in high-risk patients, in anatomically inaccessible lesions, or when symptoms are absent [7,31]. The choice of management should ideally follow multidisciplinary discussion, balancing the benefits of definitive excision against the potential morbidity of an extensive resection.

#### 3.4.5. Prognosis

Prognosis of mesenteric cystic lymphatic malformations largely depends on the completeness of surgical excision, as multiple case series and reports have demonstrated durable long-term outcomes when total resection is achieved in appropriately selected patients. For example, Saxena et al. [1] reported up to 50 months of recurrence-free survival in six adult patients who underwent either open or laparoscopic resection, while Yin et al. [32] and Li et al. [5] documented favorable results even in anatomically complex presentations, including rare sigmoid mesocolon involvement. Complete excision with histologically clear margins remains the most reliable predictor of favorable prognosis, although recurrence is still possible due to the underlying pathophysiology of lymphatic malformations [1,29,30]. Surgical intervention is generally indicated in cases of progressive enlargement, hemorrhage, or torsion risk [7,31].

#### 3.4.6. Minimally Invasive Versus Open Surgery

Minimally invasive techniques, such as laparoscopic and robotic-assisted resections, have shown promising results, particularly in pediatric and elective adult cases with well-circumscribed lesions [1,3]. However, in emergency settings or when dense adhesions compromise visualization and safe dissection, open surgery often provides a safer and more controlled approach. In situations where complete excision is not feasible without significant collateral damage, options such as image-guided sclerotherapy (e.g., with OK-432, bleomycin, or doxycycline), cyst drainage, or closure with mattress sutures reinforced with surgical glue may be considered following multidisciplinary consultation. Fenestration and marsupialization should generally be avoided, as they not only carry a high recurrence risk but can also lead to persistent lymphatic or chylous leakage, which may result in life-threatening complications [30]. Rarely, spontaneous regression of MCLMs has been reported, supporting an observational approach in carefully selected asymptomatic patients [32,33].

To aid individualized surgical planning in both pediatric and adult patients, Table 3 provides a comparative summary of laparoscopic versus open approaches, highlighting key operative and perioperative considerations—such as lesion characteristics, anatomical complexity, comorbidities, and urgency of presentation—that may guide the choice of surgical technique.

#### 3.4.7. Recent Literature and Technique Selection

Recent literature (2019–2025) underscores the importance of tailoring treatment to the individual, taking into account patient age, lesion size, anatomical complexity, comorbidities, and surgical risk. While complete excision remains an effective option for many patients, it should be pursued within a multidisciplinary framework that also considers conservative and minimally invasive alternatives, as summarized in Table 4.

Minimally invasive approaches, including laparoscopic and robotic-assisted resections, have shown increasing value in managing mesenteric lymphatic malformations (MLMs), particularly in pediatric and anatomically favorable cases [3,5]. Robotic surgery, in particular, offers enhanced visualization, precision, and potentially reduced perioperative morbidity. However, the choice of surgical technique should be guided by lesion-specific factors such as size, location, vascular involvement, and patient risk profile. Larger cysts in the jejunal mesentery have been linked to a higher risk of intraoperative complications, sometimes necessitating more extensive resections [3]. While minimally invasive methods can reduce postoperative pain and recovery time, open surgery remains essential in emergencies, in the presence of dense adhesions, or when safe laparoscopic access is not feasible.

#### 3.4.8. When Complete Excision Is Not Feasible

When complete excision is not possible without significant collateral damage, management options include image-guided sclerotherapy (e.g., OK-432, bleomycin, doxycycline), cyst drainage, or closure with mattress sutures reinforced with surgical glue, ideally after multidisciplinary consultation. Rarely, spontaneous regression of MCLMs has been reported, supporting observation in carefully selected asymptomatic cases [34].

#### 3.4.9. Intraoperative Decision-Making Principles

Intraoperative decision-making should be guided by a careful assessment of lesion characteristics, vascular relationships, and patient stability, ideally within a multidisciplinary framework. When a lesion is densely adherent or compromises bowel vascularity, segmental resection may be indicated; however, if complete excision cannot be achieved safely, alternative strategies such as image-guided sclerotherapy (OK-432, bleomycin, doxycycline), cyst drainage, or closure with mattress sutures reinforced by surgical glue can be considered in consultation with an expert center. Fenestration and marsupialization should generally be avoided, as they carry a high risk of recurrence and may cause persistent lymphatic or chylous leakage, potentially leading to life-threatening complications [30].

When feasible, postoperative MRI may be valuable to delineate the full extent of residual or multifocal lesions and to guide further management.

A stepwise intraoperative framework is outlined in Table 5, which summarizes practical decision-making options depending on cyst location, vascular involvement, and surgical feasibility.

#### 3.4.10. Histologic Classification and Diagnosis

Histologically, lymphatic malformations are classified into simple, cavernous, or cystic subtypes. Definitive diagnosis relies on histopathologic evaluation supplemented by immunohistochemical markers such as Prox1, CD31, and D2-40, which confirm lymphatic endothelial origin [35,36]. These lesions typically lack cytologic atypia or mitotic activity, supporting their benign nature [37,38]. Among mesenteric cysts—rare entities with an estimated incidence of approximately 1 per 100,000 hospital admissions—lymphatic malformations are congenital in origin and most commonly involve the jejunal and ileal mesentery [1,32].

#### 3.4.11. Clinical Presentation and Complications

Although many are asymptomatic and discovered incidentally, mesenteric lymphangiomas can become clinically significant with increasing size or when complicated by hemorrhage, infection, or bowel obstruction [1]. Macrocystic lymphatic malformations (previously referred to as cystic hygromas) may be identified prenatally via second-trimester ultrasound and are often associated with genetic syndromes such as Turner, Down, and Noonan [39]. While no prenatal data were available in the present case, a congenital origin remains likely, supporting genetic evaluation in pediatric or atypical presentations.

#### 3.4.12. Summary of Reported Cases and Case Relevance

A review of 17 reported cases of mesenteric lymphatic malformations revealed 10 male and 7 female patients, with ages ranging from 5 to 80 years (mean ≈45 years). The jejunal and ileal mesentery were the most frequent sites of involvement (10/17, 59%), followed by colonic (1/17), hepatobiliary (1/17), gastric/pancreatic (2/17), and unspecified mesenteric localizations (3/17). Cyst size varied from 3.5 to 30 cm, with most lesions exceeding 10 cm (11/17, 65%). Surgical management consisted predominantly of complete excision (10/17) or segmental enterectomy (7/17). These findings confirm that while MCLMs are rare and heterogeneous in presentation, surgical resection remains the mainstay of therapy, tailored to lesion size, anatomical location, and associated complications(Table 6).

To our knowledge, this represents one of the few reported cases documenting the coexistence of acute appendicitis and mesenteric lymphatic malformations in an adult. Management was guided by a structured intraoperative algorithm that allowed adaptation to the specific findings and ensured a balance between definitive treatment and surgical safety. Unlike most previous reports, which emphasize preoperative diagnosis or pediatric presentations, this case highlights real-time surgical decision-making and offers a practical framework for emergency surgical teams when faced with unexpected mesenteric cystic lesions.

#### 3.4.13. Pathology, Genetic Basis, and Multidisciplinary Management

Current evidence suggests that mesenteric lymphatic malformations arise from somatic genetic mutations leading to mosaicism, which explains their potential for recurrence even after apparently complete excision [14]. This genetic basis underlines the need for individualized treatment within a multidisciplinary framework that includes surgeons, interventional radiologists, pathologists, and, when appropriate, pediatric specialists [1,33]. While complete surgical excision remains the most effective treatment when feasible and safe, non-surgical modalities such as image-guided sclerotherapy may be appropriate in selected high-risk or anatomically complex cases [49,50,51,52]. In acute presentations, surgery should be pursued when symptomatic and technically feasible without unacceptable collateral damage. Post-emergency MRI may further assist in delineating the extent of disease and planning subsequent management in collaboration with specialized centers [33,50,51,52,53,54]. Optimal outcomes are therefore achieved through case-by-case decision-making that balances the benefits of definitive excision against the risks associated with more extensive procedures.

### 3.5. Case Contribution and Surgical Challenges

This case adds to the existing literature in several clinically relevant ways. First, it documents a rare concurrence of acute appendicitis and an incidental mesenteric cystic lymphatic malformation—an unusual combination with only a few cases reported to date. Second, it highlights the importance of real-time intraoperative decision-making in emergency settings, where anatomical constraints, vascular considerations, and the patient’s overall condition may influence the feasibility of complete excision. In scenarios where dense adhesions are present or critical structures are at risk, more extensive resections, such as segmental bowel removal, may be necessary, whereas in other situations, less invasive or conservative options could be appropriate. Third, the case provides an educational contribution by outlining a structured, stepwise intraoperative management strategy that underscores the importance of surgical adaptability and anatomical assessment—particularly valuable for junior surgeons and trainees facing acute surgical scenarios.

In our case, surgical excision was performed through a limited midline laparotomy following initial exploration, as this was considered the safest option given the intraoperative findings. While open surgery offers direct access and facilitates complete removal in complex cases, it is also associated with a higher risk of postoperative complications such as wound infection or incisional hernia. In selected cases, laparoscopic exploration may help define the optimal surgical plan, enabling targeted incision placement, minimizing operative trauma, and potentially reducing the risk of postoperative abdominal wall complications.

### 3.6. Limitation of Follow-Up

Although complete surgical excision was achieved and the postoperative course was uneventful, extended follow-up imaging at 3 or 6 months could not be performed because the patient was lost to follow-up after discharge. Despite repeated attempts—including scheduled outpatient appointments and telephone reminders—no post-discharge evaluation was completed. This represents a limitation of the present case, as it prevents definitive assessment of long-term recurrence risk. Nevertheless, the intraoperative findings and histopathological confirmation of resection margins suggest a favorable prognosis. Even so, the absence of follow-up underscores the importance of ongoing surveillance in similar cases, regardless of the perceived completeness of excision.

## 4. Conclusions

This case highlights the unusual intraoperative coexistence of acute appendicitis and a mesenteric cystic lymphatic malformation in an adult patient—a combination rarely reported in the literature. Beyond its diagnostic rarity, it illustrates the clinical and technical challenges posed by unexpected lymphatic lesions encountered during emergency procedures, where the management strategy must be adapted to the specific anatomical and patient-related factors present at the time of surgery.

By shifting the emphasis from preoperative diagnosis to real-time intraoperative decision-making, this article presents a structured surgical algorithm intended for use in emergent settings. The framework is anatomy-driven and flexible, allowing for a range of management options—from complete excision to more conservative approaches—based on lesion characteristics, surgical risk, and multidisciplinary input. In doing so, the report contributes both a rare case and a reproducible decision-making strategy, with direct relevance to emergency surgical practice, patient safety, and clinical education.

## Figures and Tables

**Table 1:** Clinical Timeline of Patient Presentation and Management.

Day	Clinical Event
**Day 0**	Onset of right lower quadrant pain, associated with nausea and vomiting.
**Day 1 (Morning)**	Worsening abdominal pain; presentation to the Emergency Department.
	Physical examination, laboratory investigations, and contrast-enhanced CT imaging performed.
**Day 1 (Afternoon)**	Decision for emergency surgical intervention; midline laparotomy performed.
**Postoperative Day 1–2**	Intravenous antibiotics administered; patient tolerated oral intake by Day 2.
**Postoperative Day 3–5**	Continued clinical improvement with no complications.
**Postoperative Day 5**	Discharged home in stable condition.

**Table 2:** Laboratory tests upon admission.

Parameter	Laborator Results	Normal Range
**White blood cell**	13 × 10^3^ cells/µL	4–10 × 10^3^ cells/µL
**Hemoglobin**	14 g/dl	13.5–17.5 g/d
**Platelet count**	340 × 10^3^ cells/µL	150–450 × 10^3^ cells/µL
**Neutrophilia**	85.14%	40–70%
**Creatinine**	1 mg/dL	0.6–1.3 mg/dL
**Urea**	100 mg/dL	15–45 mg/dL
**INR**	1.3	0.8–1.2
**C-reactive protein**	25 mg/dL	<0.5 mg/dL
**Fibrinogen**	450 mg/dL	200–400 mg/dL

Legend: INR = International Normalized Ratio.

**Table 3:** Comparative Summary of Surgical Approaches for Lymphatic Malformations.

Parameter	Laparoscopic Approach	Open Surgery
**Access to retroperitoneal cysts**	Limited exposure; may require conversion to open in complex or extensive cases	Excellent exposure for large, deep, or complex lesions
**Hemorrhage control**	Technically more challenging due to limited working space	More straightforward control, especially for major vessels
**Hospital stay duration**	Typically shorter with faster recovery	Slightly longer due to larger incision and postoperative pain
**Emergency suitability**	Feasible in selected cases with experienced team; useful for diagnostic exploration and optimal incision planning	Generally appropriate for acute presentations or when extensive resection anticipated

**Table 4:** Summary of Selected Published Cases on Mesenteric Lymphatic Malformations (2019–2025).

Author	Patient Type	Surgical Intervention	Imaging/IHC	Follow-Up	Key Findings
**Saxena** [1]	Adult	Cystectomy ± bowel resection	D2-40, CD31	~50 months	Complete resection with no recurrence
**Yin** [32]	Adult	Complete resection	CT/MRI	1 month	Large cyst (~15 cm) with chronic course over 20 years
**Li** [5]	Adult (sigmoid)	Laparoscopic fenestration and drainage	EUS, MRI, IHC	3 months	Rare sigmoid location, successfully managed laparoscopically
**Amrutha** [33]	Adult	Elective laparotomy with complete excision	Ultrasound + CECT	Uneventful recovery	Large (12 cm × 15 cm) mesenteric cyst mimicking gynecologic pathology; successful complete resection
**Yan** [3]	Pediatric	Robotic laparoscopic management	Clinical + radiological evaluation	Not reported	Cysts ≥ 7.5 cm and jejunal location correlated with higher complication risk
**Alqurashi** [4]	Adult	Segmental colectomy (8 cm) + cyst excision	CT abdomen	Uneventful postoperative recovery	Rare localization (transverse mesocolon); complete resection with good outcome

Legend: CT = computed tomography, MRI = magnetic resonance imaging, IHC = Immunohistochemistry, EUS = Endoscopic Ultrasound, CECT = contrast-enhanced computed tomography.

**Table 5:** Intraoperative Decision-Making Framework for Mesenteric Cystic Lymphatic Malformations: A Stepwise Surgical Approach.

Proposed Intraoperative Management Algorithm for Mesenteric Cystic Lymphatic Malformations
1. Proposed Intraoperative Management Algorithm for Mesenteric Lymphatic Malformations
○ Initial Assessment of the Lesion
○ Evaluate cyst location, size, and relation to adjacent bowel loops and mesenteric vessels.
2. Assess feasibility of safe dissection without compromising bowel vascularity.
○ Consider laparoscopic exploration for optimal visualization and surgical planning.
○ Feasibility of Complete Excision (Cystectomy)
3. If the lesion is well-circumscribed and separable from mesentery: → Perform laparoscopic or open complete cystectomy.
○ If moderate adhesions are present but bowel is viable: → Attempt careful blunt/sharp dissection, prioritizing preservation of vascular supply.
4. When Dense Adhesions Are Present
○ If bowel vascularity is at risk or the lesion cannot be safely separated: → Perform segmental bowel resection with primary anastomosis.
5. Alternative Approaches for High-risk Situations
○ If resection is unsafe due to risk of major collateral damage or patient **instability**: → Consider temporary measures such as sclerotherapy, cyst drainage, or closure of cyst cavity with mattress sutures and sealant
○ Avoid marsupialization/fenestration whenever possible due to risk of persistent lymphatic/chylous leakage.
6. Surgical Approach Selection
○ Laparoscopic approach: **Preferred in** stable, **elective cases with** accessible, **non-adherent** lesions; may prevent large incisions and postoperative hernias.
○ Open surgery: **Indicated in emergency settings** (**e.g.**, concurrent **appendicitis), giant** lesions, **or when adhesions** compromise safe **laparoscopic resection**.

**Table 6:** Overview of Reported Mesenteric Lymphatic Cysts: Presentation, Diagnosis, and Operative Data.

Author (Ref.)	Age at Diagnosis	Sex	Gastrointestinal Simptoms	Cystic Size	Surgical Treatment	Localization
**Prior-Rosas** [40]	47 years	Male	Abdominal pain and distension, dyspepsia	20.73 cm × 21 cm	Complete resection of the cyst	Ileum
**Aprea** [41]	67 years	Female	Distension, hyper active bowel sounds and tenderness	6 × 3.5 cm	Complete resection of the cyst and appendicectomy	Ileum
**Aprea** [41]	68 years	Male	Abdominal pain and a mass in RIF and RH	10 × 10 cm	Complete resection of the cyst and Choledochorrhaphy	Common hepatic duct
**Aprea** [41]	80 years	Male	Abdominal pain and occlusive state	8.6 × 5 cm	Complete resection	Jejunal loops and stomach
**Aprea** [41]	65 years	Male	Asymptomatic	18 × 12 cm	Complete resection	Stomach and pancreas
**Chen** [42]	27 years	Female	Abdominal pain	15 × 8 cm	Complete resection	Jejunum
**Chin** [43]	34 years	Male	Tumor in the left lower abdomen.	15 × 12 cm	Enterectomy	Jejunum
**Fundarò** [44]	45 years	Male	Ileal volvulus	3.5 × 3 cm	Complete resection of the cyst	Ileum
**Yoo** [45]	56 years	Female	Epigastric pain	8 × 8 cm	Complete resection of the cyst	Mesentery
**Tsukada** [46]	48 years	Male	Abdominal distention and pain	30 × 5 cm	Complete resection of the cyst	Small bowel mesentery
**Nagano** [47]	40 years	Male	Tenderness in the lower left quadrant.	4.3 × 4 cm	Enterectomy	Jejunum
**Jayasundara** [48]	18 years	Female	Abdominal pain and vomiting	10 × 10 cm	Enterectomy	Jejunal mesentery
**Yin** [32]	66 years	Male	Abdominal pain	20 × 10 cm	Enterectomy	Jejunal mesentery
**Khattala** [49]	12 years	Female	Palpable abdominopelvic mass	20 × 5 cm	Enterectomy	Jejunal mesentery
**Ramirez-Ortega** [50]	5 years	Male	Abdominal distension	18 × 11 cm	Complete resection of the cyst and sigmoidectomy	Sigmoid colon
**Mahfoud** [51]	63 years	Female	Pelvic Pain	7 × 6 cm	Enterectomy	Jejunal mesentery
**Siddique** [21]	24 years	Male	Tenderness over the left lower abdomen	20 × 16 cm	Enterectomy	Jejuno-ileal junction.

## Data Availability

The data presented in this study are available on request from the corresponding author. The data are not publicly available due to patient confidentiality.
